# Oncological outcomes of conversion therapy in gastric cancer patients with peritoneal metastasis: a large-scale retrospective cohort study

**DOI:** 10.1007/s10120-023-01452-8

**Published:** 2023-12-24

**Authors:** Zhongyin Yang, Sheng Lu, Min Shi, Hong Yuan, Zhenqiang Wang, Zhentian Ni, Changyu He, Yanan Zheng, Zhenglun Zhu, Wentao Liu, Xuexin Yao, Jun Zhang, Chen Li, Min Yan, Chao Yan, Zhenggang Zhu

**Affiliations:** 1 Shanghai Key Laboratory of Gastric Neoplasms, Department of General Surgery, Shanghai Institute of Digestive Surgery, Ruijin Hospital, Shanghai Jiao Tong University School of Medicine, No. 197 Ruijin er Road, Shanghai, China; 2 Department of Oncology, Ruijin Hospital, Shanghai Jiao Tong University School of Medicine, Shanghai, China

**Keywords:** Gastric cancer, Peritoneal metastasis, Intraperitoneal infusion, Conversion surgery, Oncological outcome

## Abstract

**Background:**

Data on the long-term oncological outcomes of patients who undergo conversion surgery (CS) in gastric cancer (GC) patients with peritoneal metastasis (PM) are limited.

**Methods:**

GC patients with PM who received intraperitoneal (ip) and systemic chemotherapy between April 2015 and January 2021 were enrolled. Multivariate analysis was performed to identify risk factors associated with survival. Clinicopathological and survival outcomes were compared between those with CS and those without CS (NCS). The paclitaxel (PTX) plus tegafur–gimeracil–oteracil potassium capsules (S-1) (PS) + ip PTX and oxaliplatin plus S-1 (SOX) + ip PTX groups were matched in a 1:1 ratio using propensity score matching. Oncological and survival data were collected and analyzed.

**Results:**

A total of 540 patients who received ip chemotherapy via subcutaneous port and systemic chemotherapy were analyzed and 268 patients were enrolled, including 113 who underwent CS and 155 who did not. Overall survival (OS) were 27.0 months and 11.8 months in the CS and NCS groups (*P* < 0.0001), respectively. R0 resection was an independent prognostic factor for patients who underwent CS. The OS of patients with or without ovariectomy was 21.3 or 12.0 months (*P* < 0.0001). No difference of clinicopathological and survival outcomes was found between the PS + ip PTX and SOX + ip PTX groups.

**Conclusion:**

Conversion therapy is safe and adverse events were manageable. CS improves the survival of GC patients with PM after ip and systemic chemotherapy. R0 is an important prognostic factor. Furthermore, outcomes are comparable between the PS + ip PTX and SOX + ip PTX groups.

**Supplementary Information:**

The online version contains supplementary material available at 10.1007/s10120-023-01452-8.

## Introduction

Gastric cancer (GC) is a prevalent malignant disease worldwide and is a leading cause of mortality, particularly in China where a large proportion of patients were diagnosed at an advanced stage [1, 2]. Peritoneal metastasis (PM) is the most frequent mode of metastasis in GC, and is associated with an extremely poor prognosis [3, 4]. However, the therapeutic mode for GC with PM remains limited.

The REGATTA clinical trial showed that palliative gastrectomy followed by chemotherapy for advanced GC with a single non-curable factor such as PM did not improve survival outcomes [5]. Despite recent advances in systemic chemotherapy, the median survival time of patients receiving systemic chemotherapy is only 3.1–14.1 months [6–8]. Cytoreductive surgery combined with hyperthermic intraperitoneal chemotherapy and pressurized intraperitoneal aerosol chemotherapy have demonstrated efficacy with limited survival time in specialized centers for the treatment of GC patients with PM [9–13]. Therefore, for the GC patients with PM, conversion therapy based on comprehensive treatment has been attempted to reduce the stage of the primary tumor and eliminate PM, thereby providing an opportunity for radical resection in selected patients.

However, focusing on conversion therapy in patients with PM, long-term oncological and prognostic outcomes remain unknown. Although the PHOENIX clinical trial has demonstrated the efficacy and safety of intravenous infusion of paclitaxel (PTX) and oral tegafur-gimeracil-oteracil potassium capsules (S-1) with ip PTX (PS + ip PTX) in patients with PM, conversion surgery (CS) outcomes have not been fully elucidated [14]. The oxaliplatin plus S-1 (SOX) with intraperitoneal PTX (SOX + ip PTX) regimen also showed efficacy in some patients with PM [15, 16]. In addition, several studies have demonstrated survival benefits in pretreated patients after CS [17–22]. However, these reports were limited by a small number of patients and short follow-up periods.

In this retrospective study, we provide detailed data from a large-scale cohort on the oncological and survival outcomes of patients who received intraperitoneal infusion PTX-based conversion therapy and compared the clinicopathological outcomes and prognosis between the PS + ip PTX and SOX + ip PTX regimens.

## Methods

### Patients

Adult patients diagnosed of GC with PM who received intra-abdominal port placement and PS + ip PTX or SOX + ip PTX regimens between April 2015 and January 2021 in Ruijin Hospital affiliated with the Shanghai Jiao Tong University School of Medicine were enrolled in this study. All patients met the following criteria: (1) 18–85 years of age, (2) PM from GC diagnosed via laparoscopy and subsequently confirmed through various diagnostic methods, including biopsy of suspected lesions, cytological examination of ascites, and/or lavage cytology, (3) without gastric outflow tract obstruction or intestinal obstruction, (4) Eastern Cooperative Oncology Group (ECOG) score ≤ 2, (5) expected life expectancy ≥ 3 months, and (6) adequate organ function. The exclusion criteria were as follows: (1) distal metastasis(es) except ovary (e.g. lymph nodes except regional lymph node(s), liver, lung, bone, brain, etc.), (2) prophylactic port placement, (3) positive peritoneal cytology without visible PM, (4) peritoneal recurrence after gastrectomy, (5) patients received systemic chemotherapy without intraperitoneal chemotherapy, (6) patients enrolled in another phase III clinical trial, or (7) loss to follow-up. This study was approved by the ethics committee of Ruijin Hospital affiliated to Shanghai Jiao Tong University, School of Medicine, all patients signed informed consents before the operation.

### Treatment

Laparoscopic exploration confirmed PM in all patients, and intra-abdominal ports were implanted in the subcutaneous space of the lower abdomen. Subsequently, the patients received treatment every 3 weeks as follows: PS + ip PTX regimen consisting of intravenous paclitaxel (50 mg/m^2^), intraperitoneal paclitaxel (20 mg/m^2^) on days 1 and 8, and oral S-1 (80 mg/m^2^) on days 1–14 or SOX + ip PTX regimen consisting of intravenous oxaliplatin (100 mg/m^2^) on day 1, intraperitoneal paclitaxel (40 mg/m^2^) on days 1 and 8 and oral S-1 (80 mg/m^2^) on days 1–14 [15, 23].

The evaluation was performed every three cycles, cytological examination was performed in the first laparoscopic examination with ascites or peritoneal lavage fluid, and before the second-look laparoscopy with ascites or peritoneal lavage fluid collected through an intraperitoneal port. The criteria for the second-look laparoscopy was set as follows: (1) disappearance or remarkable shrinkage of peritoneal metastasis by imaging, (2) negative peritoneal cytology, (3) no other distant metastasis, (4) downstaging of the primary tumor, (5) the patient’s general condition improved. Gastrectomy with D2 lymphadenectomy was performed for patients who met the criteria as follows: (1) disappearance or remarkable shrinkage of peritoneal metastasis by second-look laparoscopy, (2) negative peritoneal cytology, (3) no other distant metastasis, (4) downstaging of the primary tumor, (5) the patient’s general condition improved. Remarkable shrinkage of peritoneal metastasis was determined to be the isolated remnant lesion or lesion size less than 5 cm [23]. Owing to the preoperative confirmation of negative cytology, R0 resection is defined as the complete removal of visible tumors with negative tumor margins, R1 resection is defined as patients have microscopic residual tumor, whereas R2 resection refers to the macroscopic residual of tumor after surgery. After CS the primary regimens were continued until intolerable toxicity or disease progression.

### Data collection

Patient demographic data including sex, age, body mass index (BMI), ECOG performance status, PM, amount of ascites, pathological grading, peritoneal cancer index (PCI), carcinoembryonic antigen (CEA), serum carbohydrate antigens 125 (CA125) and 19-9 (CA 19-9), history of chemotherapy, ovarian metastasis (female), surgical type, extent of resection, and combination of resections were collected. Pathological characteristics after CS included tumor size, primary tumor location, tumor gross type, resected lymph nodes, and mismatch repair status. Short-term postoperative outcomes including common complications (intra-abdominal hemorrhage, wound infection, anastomotic leakage, and abdominal infection), length of hospital stay, and 30-day morbidity were recorded in detail. Pathological response was defined as residual tumor cells with tumor regression grade (TRG) according to Becker criteria [24].

Oncological outcomes included overall survival (OS) and progression-free survival (PFS), which were defined as the time from diagnosis to death from any cause or the last date of follow-up (cut-off date: January 31, 2023) and the time from diagnosis to disease progression or death from any cause in the absence of progression, respectively.

### Statistical analysis

Statistical analyses were conducted and graphs were created using R software (version 4.2.2; R Foundation for Statistical Computing, Vienna, Austria). Differences between groups were assessed using the Student's* t* test for normally distributed variables, and the rank sum test for non-normally distributed variables. The chi-squared test and Fisher's exact test (when appropriate) were used to compare categorical variables. Descriptive statistics of the clinicopathological characteristics were performed. The 95% confidence intervals (CIs) for the median survival time were calculated. Survival curves were estimated using the Kaplan–Meier method. Univariate and multivariate Cox regression analyses were used to evaluate the prognostic significance of clinicopathological factors. Statistical significance was set at *P* < 0.05. Stepwise regression was used for variable selection to identify the optimal combination of variables in the Cox regression model. To achieve this, we used the stepAIC function in R, which selects the best model from a given set of variables based on the Akaike information criterion (AIC).

In order to assess the impact of two therapy regimens on oncologic outcomes, we performed a propensity score matching (PSM) analysis. The PSM was executed using the "MatchIt" package within the R language environment, employing 1:1 optimal matching. This technique was chosen to minimize potential bias between the PS + ip PTX and SOX + ip PTX regimens. The matching process included several covariates: patient's age, sex, BMI, ECOG-PS, PCI, ascites volume, tumor location, histological type, ovarian metastasis, serum CA125, CA199 and CEA levels. After the matching process, we assessed the balance of covariates using the standardized mean difference (SMD). While it's generally ideal to aim for an SMD value less than 0.1 indicating minor imbalance, it is important to recognize that the acceptable SMD can be influenced by the context of the specific research. Therefore, we carefully considered our SMD results within the context of our study when interpreting our findings. All tests were two-tailed, and *P* < 0.05 was considered statistically significant.

## Results

### Clinical characteristics

**Fig. 1:**
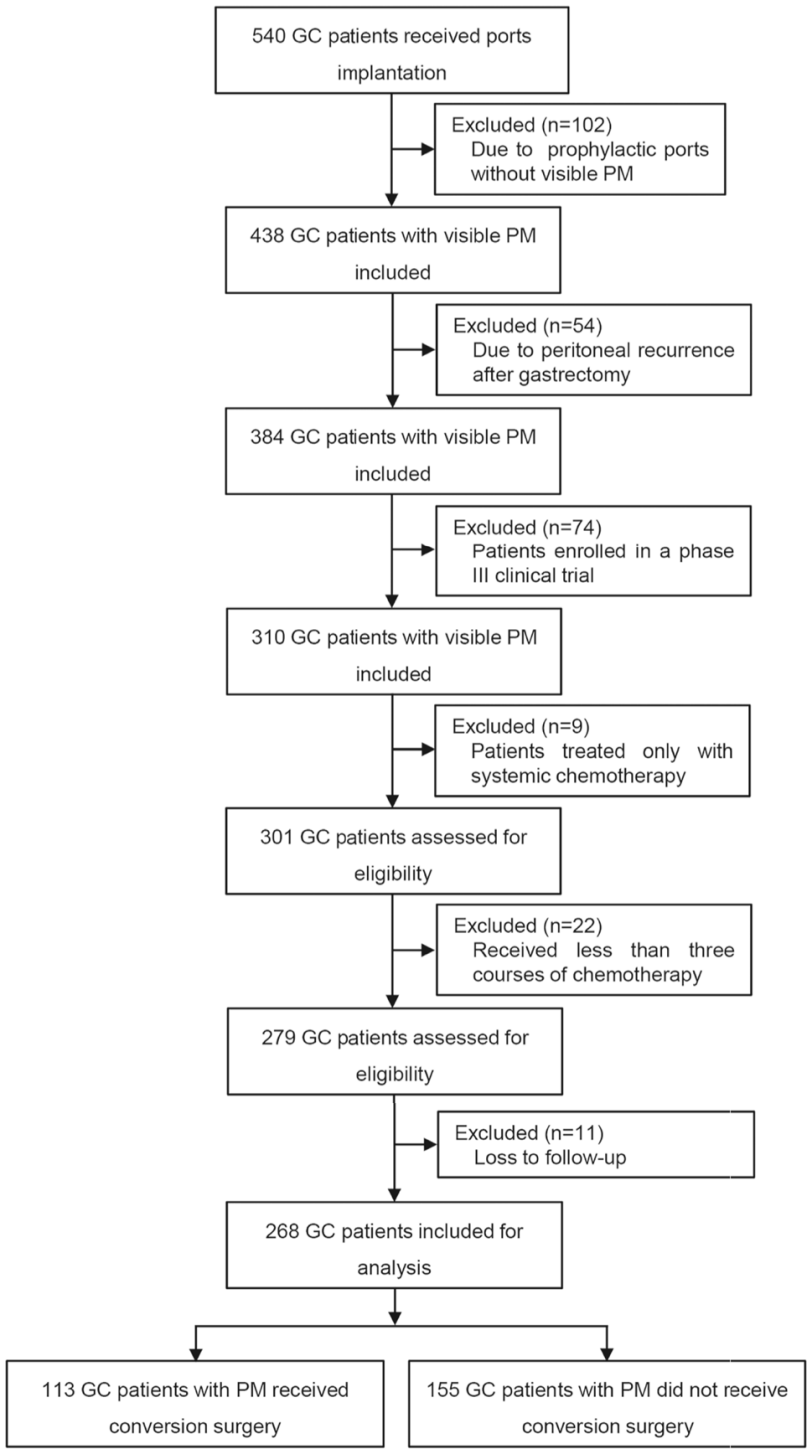
Study flowchart. *GC* gastric cancer, *PM* peritoneal metastasis

From April 2015 to January 2021, a total of 540 GC patients who underwent subcutaneous port implantation in Ruijin Hospital were retrospectively analyzed. After screening according to the inclusion and exclusion criteria, 268 GC patients with PM were enrolled in the final analysis, including 113 who underwent CS and 155 with NCS. A flow diagram describing patient enrolment, inclusion, exclusion, and follow-up is shown in Fig. 1. Patients who underwent CS had significantly smaller amounts of ascites and lower PCI scores. Moreover, the CEA, CA125 and CA19-9 levels were significantly higher in the NCS group than in the CS group. However, no significant differences were observed in history of chemotherapy, tumor location and histological type (Table 1). Eighty-one of 113 (71.7%) and 116 of 155 (74.8%) patients had positive cytology in the CS and NCS groups, respectively.

**Table 1:** Baseline clinicopathological characteristics

Variables	Total cohort		*P* Value
	CS	NCS	
Age, median (years)	49.9 (28–76)	51.9 (20–85)	0.2253
Sex, *n* (%)			0.0588
Male	43 (16.0%)	77 (28.7%)	
Female	70 (26.1%)	78 (29.1%)	
BMI, kg/m^2^, *n* (%)			0.2423
> 20	84 (31.3%))	105 (39.2%)	
≤ 20	29 (10.8%)	50 (18.7%)	
ECOG-PS, *n* (%)			0.5192
0	40 (14.9%)	51 (19.0%)	
1	64 (23.9%)	85 (31.7%)	
2	9 (3.4%)	19 (7.1%)	
Peritoneal metastasis, *n* (%)			0.1536
P1a	17 (6.3%)	13 (4.9%)	
P1b	23 (8.6%)	27 (10.1%)	
P1c	73 (27.2%)	115 (42.9%)	
Amount of ascites, mL, *n* (%)			0.0087
≤ 100	62 (23.1%)	60 (22.4%)	
> 100	51 (19.0%)	95 (35.4%)	
PCI score, *n* (%)			< 0.0001
0–9	28 (10.4%)	22 (8.2%)	
10–25	63 (23.5%)	61 (22.8%)	
26–39	22 (8.2%)	72 (26.9%)	
Tumor location			0.8201
U	12 (4.5%)	20 (7.5%)	
M	74 (27.6%)	101 (37.7%)	
L	27 (10.1%)	34 (12.7%)	
Histological type			0.4399
tub2	5 (1.8%)	3 (1.1%)	
por	94 (35.1%)	124 (46.3%)	
sig	12 (4.5%)	23 (8.5%)	
muc	2 (0.7%)	5 (1.9%)	
CA125 at diagnosis, Median (IQR), U/ml	35.7 (16.7, 102.2)	64.0 (26.7, 198.0)	0.0004
CA19-9 at diagnosis, Median (IQR), U/ml	14.3 (6.5, 45.1)	13.3 (5.0, 60.1)	< 0.0001
CEA at diagnosis, Median (IQR), ng/mL	1.67 (1.02, 3.06)	2.15 (1.19, 4.99)	0.0165
History of chemotherapy, *n* (%)			0.0992
With	39 (14.6%)	69 (25.7%)	
Without	74 (27.6%)	86 (32.1%)	
Ovarian metastasis, Female cohort, *n* (%)			0.1804
With	23 (15.5%)	34 (23.0%)	
Without	47 (31.8%)	44 (29.7%)	

### Adverse events

During preoperative treatment, the most common chemotherapy-related adverse events were leukopenia, neutropenia, and anemia. Most of these hematological and non-hematological toxicities were below grade 3 and were tolerable (Table 2). Intraperitoneal port related complications were observed in 60 (22.4%) patients, which was comparable to the previous reports [25, 26]. The most common complications were subcutaneous liquid accumulation (24/60, 40%) and infection (17/60, 28.3%). In addition, rotation of the port body (8 patients), wound dehiscence (8 patients), inflow obstruction (2 patients), and subcutaneous metastasis (1 patients) were also observed; however, most of these complications were grade 1 or 2, and there were no treatment-related deaths or unexpected serious adverse events (Table 2).

**Table 2:** Adverse events in all patients

Adverse Events	Grade 1		Grade 2		Grade 3		Grade 4	
	No	%	No	%	No	%	No	%
Chemotherapy-related events								
Leukopenia	71	26.5	57	21.3	30	11.2	3	1.1
Neutropenia	61	22.8	54	20.1	32	11.9	6	2.2
Anemia	139	51.9	62	23.1	24	9.0	4	1.5
Thrombocytopenia	35	13.1	13	4.9	3	1.1	0	0.0
ALT increased	49	18.3	28	10.5	4	1.5	0	0.0
AST increased	67	25.0	31	11.6	5	1.9	0	0.0
Creatinine increased	18	6.7	5	1.9	0	0.0	0	0.0
Fatigue	92	34.3	28	10.5	0	0.0	0	0.0
Nausea	51	19.0	16	6.0	3	1.1	0	0.0
Vomiting	26	9.7	7	2.6	0	0.0	0	0.0
Diarrhea	47	17.5	18	6.7	3	1.1	0	0.0
Anorexia	74	27.6	28	10.5	8	3.0	0	0.0
Peripheral neuropathy	52	19.4	18	6.7	2	0.8	0	0.0
Alopecia	76	28.4	33	12.3	NA	NA	NA	NA
Fever	39	14.6	20	7.5	1	0.4	0	0.0
Port-related events								
Infection	0	0	4	1.5	4	1.5	9	3.4
Port rotation	5	1.9	2	0.8	0	0	1	0.4
Wound dehiscence	1	0.4	4	1.5	1	0.4	2	0.8
Inflow obstruction	0	0	0	0	1	0.4	1	0.4
Subcutaneous accumulation	16	6.0	3	1.1	2	0.8	3	1.1
Subcutaneous metastasis	0	0	0	0	0	0	1	0.4

NCI-CTCAE version 5.0

*AST* Aspartate transaminase, *ALT* alanine transaminase

### Outcomes of CS

All patients received a median of six courses (range 3–12 courses) of pre-operative chemotherapy. Of the 268 patients, 264 exhibited measurable disease based on imaging, which included 93 (35.2%) individuals demonstrating partial response (PR), 145 (54.9%) individuals exhibiting stable disease (SD), and 26 (9.8%) individuals presenting progressive disease (PD) according to the World Health Organization (WHO) criteria. All the patients in the CS group had a reverted negative cytology, while peritoneal cytology reverted to negative in 84 (72.4%) of 116 patients in the NCS group. In total, 125 patients met the criteria for second-look investigative laparoscopy and 113 successfully underwent CS. Total or distal gastrectomy was performed in 80 (70.8%) or 33 (29.2%) patients, respectively. The median operative time was 240 min (range 126–427 min), and median blood loss was 60 mL (range, 20–800 mL). R0 resection was performed in 90 patients (79.6%); however, 23 patients who showed obvious shrinkage of the PM did not have all visible nodules removed (R2). Ovariectomy was performed in 23 patients with ovarian metastasis, cholecystectomy in four patients, distal pancreatectomy and splenectomy in two patients, and partial colectomy in two patients. The median number of metastatic lymph nodes was 5 (range 1–32). Six patients exhibited deficient mismatch repair (dMMR; Supplementary Table S1).

The median length of postoperative stay was 12 days (range 7–82 days). Postoperative complications, such as bleeding, leakage, bowel obstruction, and infection, were observed in 25 patients (22.1%); however, most were in grades 1 and 2, and only one patient died due to bleeding (Supplementary Table S2).

### Oncological outcomes

**Fig. 2:**
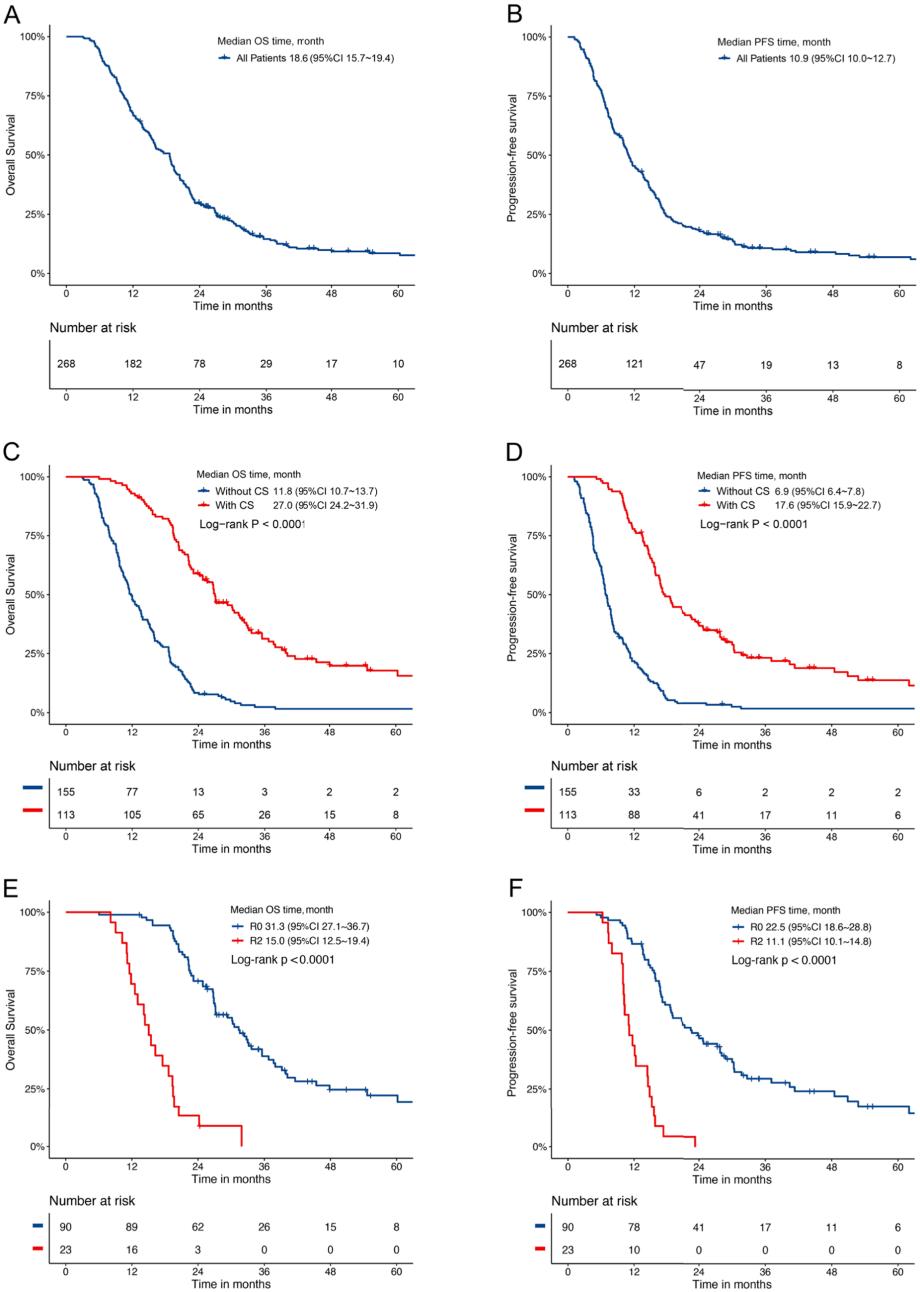
Kaplan–Meier curve of median OS and PFS of patients in the entire cohorts (**A**, **B**). Kaplan–Meier curve of median OS and PFS of patients with and without CS (**C**, **D**). Kaplan–Meier curve of median OS and PFS of patients who underwent R0 and R2 resection (**E**, **F**)

During a median follow-up time of 54.5 months (3–89.6 months), the median OS was 18.6 months (95% CI, 15.7–19.4) and the median PFS was 10.9 months (95%CI, 10.0 ~ 12.7) in the entire group (Fig. 2A, B). However, patients in the CS group had a significantly longer median OS and PFS than those in the NCS group (27.0 vs. 11.8 months, *P* < 0.0001 and 17.6 vs. 6.9 months, *P* < 0.0001; Fig. 2C, D). In particular, the median OS for patients who underwent R0 and R2 resection was 31.3 and 15.0 months (*P* < 0.0001), respectively, and the PFS for those who underwent R0 and R2 resection was 22.5 and 11.1 months (*P* < 0.0001), respectively (Fig. 2E, F). Meanwhile, OS and PFS were not significantly different between patients with and without previous chemotherapy (27.1 vs. 26.7 months, *P* = 0.52 and 22.5 vs. 16.6 months, *P* = 0.22; Supplementary Figure S1). Furthermore, patients with Borrmann type IV cancer had worse OS and PFS than those with other types of cancer (*P* < 0.01; Supplementary Figure S2A, B). However, favorable OS and PFS were observed in patients with a lower metastatic lymph node ratio (LNM) and TRG (*P* < 0.01; Supplementary Figure S2C-F).

**Fig. 3:**
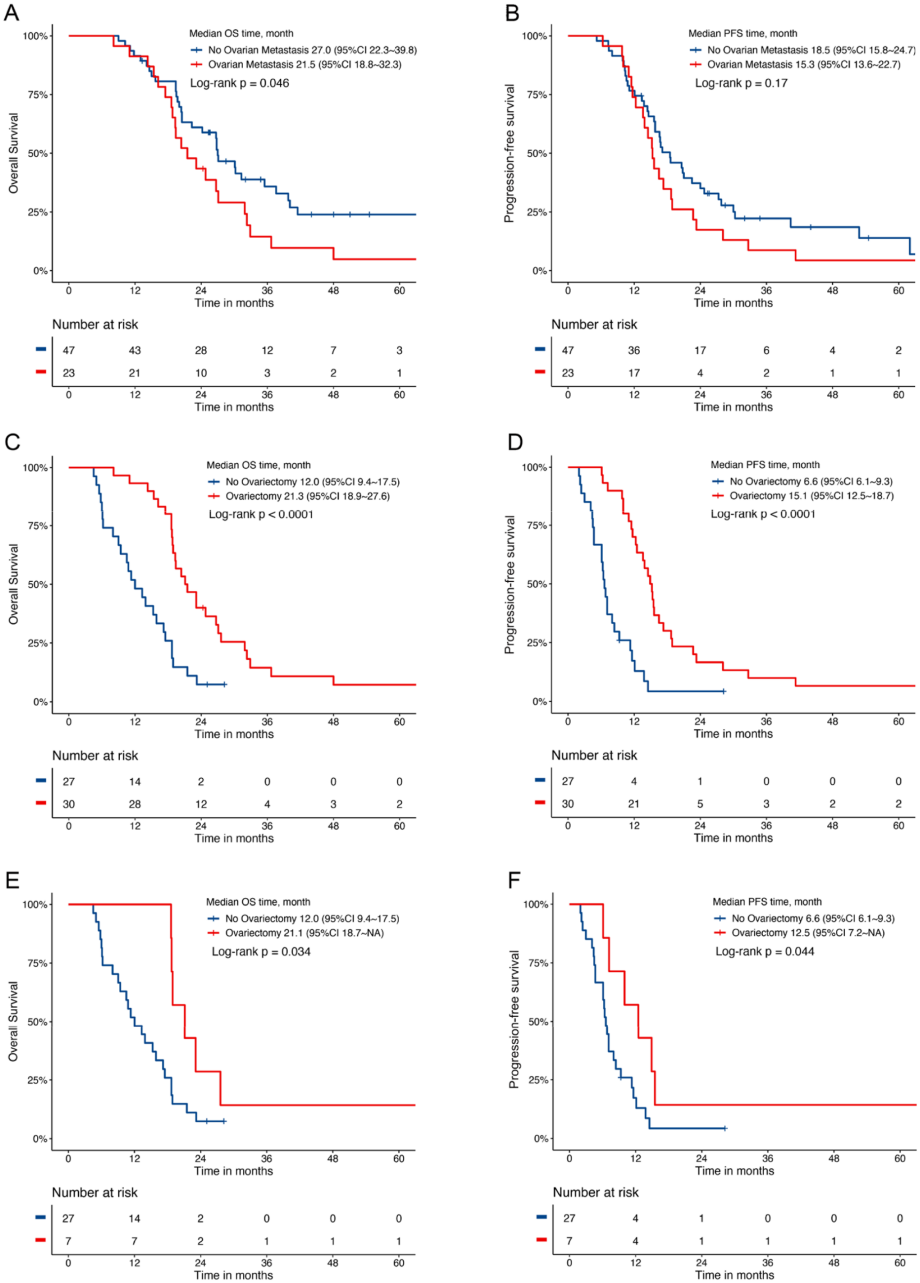
Kaplan–Meier curve of median OS and PFS of female patients with and without ovarian metastasis in the CS group (**A**, **B**). Kaplan Meier curve of median OS and PFS of female patients with and without ovariectomy (**C**, **D**). Kaplan–Meier curve of median OS and PFS of female patients in the NCS group with or without ovariectomy (**E**, **F**)

In addition, female patients without ovarian metastasis had longer OS than those with ovarian metastasis in the CS group (27.0 vs. 21.5 months, *P* = 0.046; Fig. 3A, B). In total, 57 patients had ovarian metastasis, including 30 who underwent ovariectomy and 27 who did not; the results showed that survival was better in the ovariectomy group than in non-ovariectomy group (OS: 21.3 vs 12.0 months, *P* < 0.0001; PFS: 15.1 vs 6.6 months, *P* < 0.0001; Fig. 3C, D). In the NCS group, 34 female patients had ovarian metastasis, of which seven patients underwent ovariectomy despite not having undergone CS, and the prognosis of female patients with ovarian metastasis could also benefit from ovariectomy (OS: 21.1 vs 12.0 months, *P* = 0.034; PFS: 12.5 vs 6.6 months, *P* = 0.044; Fig. 3E, F).

**Fig. 4:**
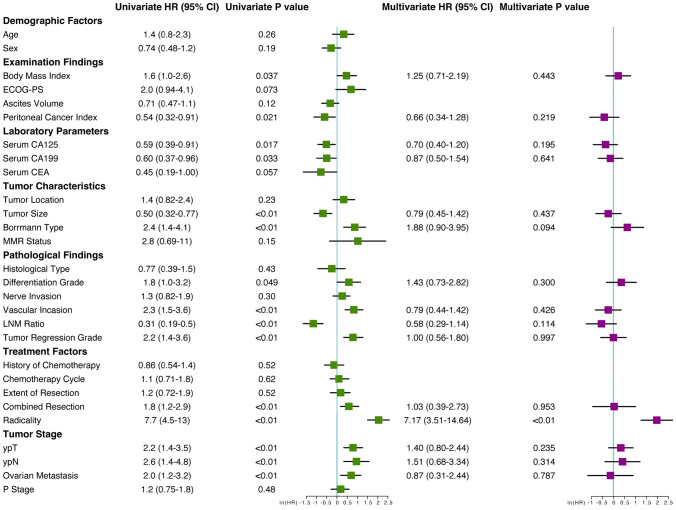
Forest plot of clinicopathological variables in the univariate and multivariate analysis

Having determined that patients who underwent CS had significantly better survival than NCS patients, further univariate and multivariate COX regression analyses were performed to explore the independent prognostic factors for CS survival. As indicated in Fig. 4, radicality (R0 resection) was a significant independent predictor of better survival in GC patients with PM.

### Comparison of the PS + ip PTX and SOX + ip PTX regimens

In this study, 35 patients received the SOX + ip PTX regimen, and we compared the PS + ip PTX and SOX + ip PTX regimens in terms of both oncological and survival outcomes. In the initial analysis, there were no differences in the entire cohort before and after PSM (Supplementary Table S3 and Figure S3A, B); however, in the CS cohort, the tumor size in the SOX + ip PTX group was significantly reduced compared to that in the PS + ip PTX group (*P* = 0.043), whereas the other clinicopathological characteristics and survival outcomes were comparable (Table 3 and Supplementary Figure S3C, D). However, after PSM, no significant differences were found between the two groups in terms of clinicopathological characteristics and survival times (Table 3 and Supplementary Figure S3E, F).

**Table 3:** Clinicopathological characteristics for PS + ip PTX and SOX + ip PTX regimens in the CS group, before and after propensity score matching

Variables	Conversion surgery cohort				Propensity score matching cohort			
	PS + ip PTX (*n* = 95)	SOX + ip PTX (*n* = 18)	*P* Value	SMD	PS + ip PTX (*n* = 18)	SOX + ip PTX (*n* = 18)	*P* Value	SMD
Age, mean (SD)	49.46 (12.52)	52.17 (14.11)	0.412	0.203	51.11 (13.74)	52.17 (14.11)	0.821	0.076
Sex, *n* (%)			0.382	0.290			1.000	0.111
Male	34 (35.8)	9 (50.0)			8 (44.4)	9 (50.0)		
Female	61 (64.2)	9 (50.0)			10 (55.6)	9 (50.0)		
BMI, mean (SD)	22.12 (3.49)	22.22 (3.25)	0.908	0.031	22.14 (5.92)	22.22 (3.25)	0.961	0.017
ECOG-PS, mean (SD)	0.73 (0.61)	0.72 (0.57)	0.979	0.007	0.67 (0.59)	0.72 (0.57)	0.777	0.095
Preoperative courses, mean (SD)	6.47 (2.64)	5.78 (3.93)	0.348	0.208	6.44 (2.68)	5.78 (3.93)	0.556	0.198
PCI, mean (SD)	17.38 (9.31)	14.17 (9.88)	0.186	0.335	15.50 (7.90)	14.17 (9.88)	0.657	0.149
Ascites volume, mean (SD)	517.79 (1132.77)	250.00 (372.59)	0.324	0.318	206.67 (350.73)	250.00 (372.59)	0.722	0.120
Tumor location, *n* (%)			0.983	0.048			0.403	0.461
U	10 (10.5)	2 (11.1)			3 (16.7)	2 (11.1)		
M	62 (65.3)	12 (66.7)			8 (44.4)	12 (66.7)		
L	23 (24.2)	4 (22.2)			7 (38.9)	4 (22.2)		
Type of gastrectomy, *n* (%)			1.000	0.038			0.488	0.352
Distal	28 (29.5)	5 (27.8)			8 (44.4)	5 (27.8)		
Total	67 (70.5)	13 (72.2)			10 (55.6)	13 (72.2)		
Tumor size, mean (SD)	5.35 (3.20)	3.76 (1.88)	0.043	0.608	3.28 (1.46)	3.76 (1.88)	0.400	0.284
Radicality, *n* (%)			0.457	0.299			1.000	0.202
R0	74 (77.9)	16 (88.9)			17 (94.4)	16 (88.9)		
R2	21 (22.1)	2 (11.1)			1 (5.6)	2 (11.1)		
Macroscopic type, *n* (%)			0.874	0.219			0.637	0.445
Borrmann I	3 (3.2)	1 (5.6)			2 (11.1)	1 (5.6)		
Borrmann II	5 (5.3)	1 (5.6)			0 (0.0)	1 (5.6)		
Borrmann III	70 (73.7)	14 (77.8)			15 (83.3)	14 (77.8)		
Borrmann IV	17 (17.9)	2 (11.1)			1 (5.6)	2 (11.1)		
ypT			0.382	0.535			0.934	0.123
ypT0-1	9 (9.5)	0 (0.0)			0 (0.0)	0 (0.0)		
ypT2	9 (9.5)	3 (16.7)			3 (16.7)	3 (16.7)		
ypT3	23 (24.2)	6 (33.3)			7 (38.9)	6 (33.3)		
ypT4	54 (56.8)	9 (50.0)			8 (44.4)	9 (50.0)		
ypN			0.923	0.171			0.714	0.397
ypN0	21 (22.1)	4 (22.2)			3 (16.7)	4 (22.2)		
ypN1	15 (15.8)	4 (22.2)			3 (16.7)	4 (22.2)		
ypN2	18 (18.9)	3 (16.7)			6 (33.3)	3 (16.7)		
ypN3	41 (43.2)	7 (38.9)			6 (33.3)	7 (38.9)		
TRG, *n* (%)			0.740	0.200			0.409	0.457
Grade 0–1	24 (25.3)	5 (27.8)			3 (16.7)	5 (27.8)		
Grade 2	46 (48.4)	7 (38.9)			11 (61.1)	7 (38.9)		
Grade 3	25 (26.3)	6 (33.3)			4 (22.2)	6 (33.3)		
MMR Status, *n* (%)			0.601	0.367			NA	< 0.001
dMMR	6 (6.3)	0 (0.0)			0 (0.0)	0 (0.0)		
pMMR	89 (93.7)	18 (100.0)			18 (100.0)	18 (100.0)		

## Discussion

Conversion therapy is considered as an investigative treatment for GC patients with PM [18], and the ultimate goal is to improve the oncological outcomes and prolong survival. In this article, we report the largest single-center retrospective study on conversion therapy in GC patients with PM that has been documented worldwide. Besides, this study is the first to compare the oncological and survival outcomes between patients who received the PS + ip PTX and SOX + ip PTX regimens.

The PCI score is an important factor that affects patient survival [27], and patients in the entire cohort with higher PCI scores had worse OS and PFS (Supplementary Figure S4). These results indicate that patients with higher PCI scores tend to have worse survival and more difficult to undergo CS. The detection and evaluation of peritoneal cytology in this study is through the utilization of an intraperitoneal port, which has been endorsed as effective by Japanese researchers [14]. We found that the cytology negative conversion rate among patients who initially tested positive was 83.7% (165 in 197 patients) in the entire cohort with ip chemotherapy. This outcome is largely comparable to that of the previous reports [14, 22, 23]. These findings underscore the efficacy of intraperitoneal chemotherapy in controlling peritoneal metastasis.

The role of surgical management of ovarian metastasis in GC remains controversial [28–30]. The treatment of patients with synchronous presentation of PM and ovarian metastasis is extremely difficult and lacks consensus [30, 31]. Metastasectomy combined with systemic chemotherapy has been reported to prolong the survival of patients with synchronous or metachronous Krukenberg tumors [32–34]. In this study, we found that ovarian metastasis was detrimental to the survival of female patients in the CS group and that ovariectomy might improve the survival. Most patients underwent synchronous ovariectomy which was performed in combination with gastrectomy after preoperative treatment. Moreover, in female patients with ovarian metastasis who did not undergo CS, ovariectomy also prolonged the survival time. Ovariectomy was identified as an independent predictor for better prognosis in the NCS group (Supplementary Table S4). Based on these results, we propose that ovarian metastasis with PM, if not caused serious complications, could receive chemotherapy first and subsequently undergo combined resection when performing CS; for these in the NSC group, ovariectomy may be a therapeutic option.

Advanced GC with high microsatellite instability (MSI-H)/dMMR was reported to be associated with a poorer response after platinum-based neoadjuvant chemotherapy, but its prognosis is usually better than microsatellite stable (MSS)/proficient mismatch repair (pMMR) [35, 36]. In the 113 patients who underwent CS, we found six patients with dMMR, although the numbers were small and dMMR was not an independent prognostic factor, the OS of patients with dMMR significantly improved after conversion therapy compared with that of patients with pMMR (Supplementary Figure S5). This result indicates that the PTX-based therapy may be favorable for peritoneal patients with dMMR. Considering the latest results of the Checkmate-649 clinical trial [37], concomitant treatment with PTX-based regimen with immune checkpoint inhibitors may be an attractive option for GC with PM.

In the CS group, R0 resection was an important independent prognostic factor. In the early stage, we performed CS by confirming the negative cytology and disappearance or obvious shrinkage of peritoneal metastasis, which resulted in a high proportion of R2 resection. And patients who received R2 resection had a relatively poorer survival. Based on these results, we proposed that the indication of CS should be changed to the disappearance of PM by the second-look laparoscopy. R0 resection is recommended for patients who undergoing the CS, and R2 resection should be avoided if possible. In our center, the R2 resection rate has decreased since the initial stage owing to the accumulation of clinical experience (Supplementary Figure S6).

The SOX regimen has shown significant efficacy in locally advanced and metastatic GC, especially against the primary tumors and extraperitoneal metastases [38–40]. Previous studies have showed improved survival for the patients who received this regimen [15, 41]. However, there are no reports on the difference between the PS + ip PTX and SOX + ip PTX regimens for the treatment of patients with PM. Our study found that the primary tumor size was significantly decreased in the SOX + ip PTX group. However, after PSM no differences were found indicating that the oxaliplatin-based regimen had comparable efficacy in the treatment of GC patients with PM during the short-term course of therapy. On the other hand, in most patients who underwent CS, the use of oxaliplatin was suspended after surgery because of systemic toxicity, but PTX-based regimens were administered over a long period (Supplementary Table S5).

The present study has two main limitations. First, this is a single-center study, which might cause potential bias in the outcomes, and potential differences in patient management, race and region across multi-institution could not be reflected. In addition, although our study claims comparable outcomes between the PS + ip PTX and SOX + ip PTX groups, there may still be unknown or unmeasured differences between the two groups that might influence their respective outcomes, even after PSM. This indicates a need for further investigation through randomized controlled trials to compare the efficacy of these two regimens.

## Conclusion

The results of this study indicate that conversion therapy is safe and feasible and that CS improves the survival of GC patients with PM. R0 resection is recommended when performing CS. Ovarian metastasis is an adverse prognostic factor, and ovariectomy might be beneficial to the survival of female patients. The effects of the PS + ip PTX and SOX + ip PTX regimens are comparable in the treatment of GC with PM. This study demonstrates the promising prospects of using ip PTX-based therapy with CS for GC with PM and provides a basis for prospective clinical trials.

### Supplementary Information

Below is the link to the electronic supplementary material.

Supplementary file1 (DOCX 1712 KB)
