# Supplementary material for: Oncological outcomes of conversion therapy in gastric cancer patients with peritoneal metastasis: a large-scale retrospective cohort study
Source: Gastric Cancer. 2023 Dec 24;27(2):387–99. doi: 10.1007/s10120-023-01452-8 (PMC10896904; doi:10.1007/s10120-023-01452-8)
Supplement: Supplementary file 1 — Supplementary file1 (DOCX 1712 KB) [file 10120_2023_1452_MOESM1_ESM.docx]

**Supplemental Materials for**

**Oncological outcomes of conversion therapy in gastric cancer patients with peritoneal metastasis: A large-scale retrospective cohort study**

Zhongyin Yang^1,†^, Sheng Lu^1,†^, Min Shi^2,†^, Hong Yuan^2^, Zhenqiang Wang^1^, Zhentian Ni^1^, Changyu He ^1^, Xuexin Yao^1^, Yanan Zheng^1^, Zhenglun Zhu^1^, Wentao Liu^1^, Jun Zhang^2^, Chen Li^1^, Min Yan^1^, Chao Yan^1,*^ , Zhenggang Zhu^1^


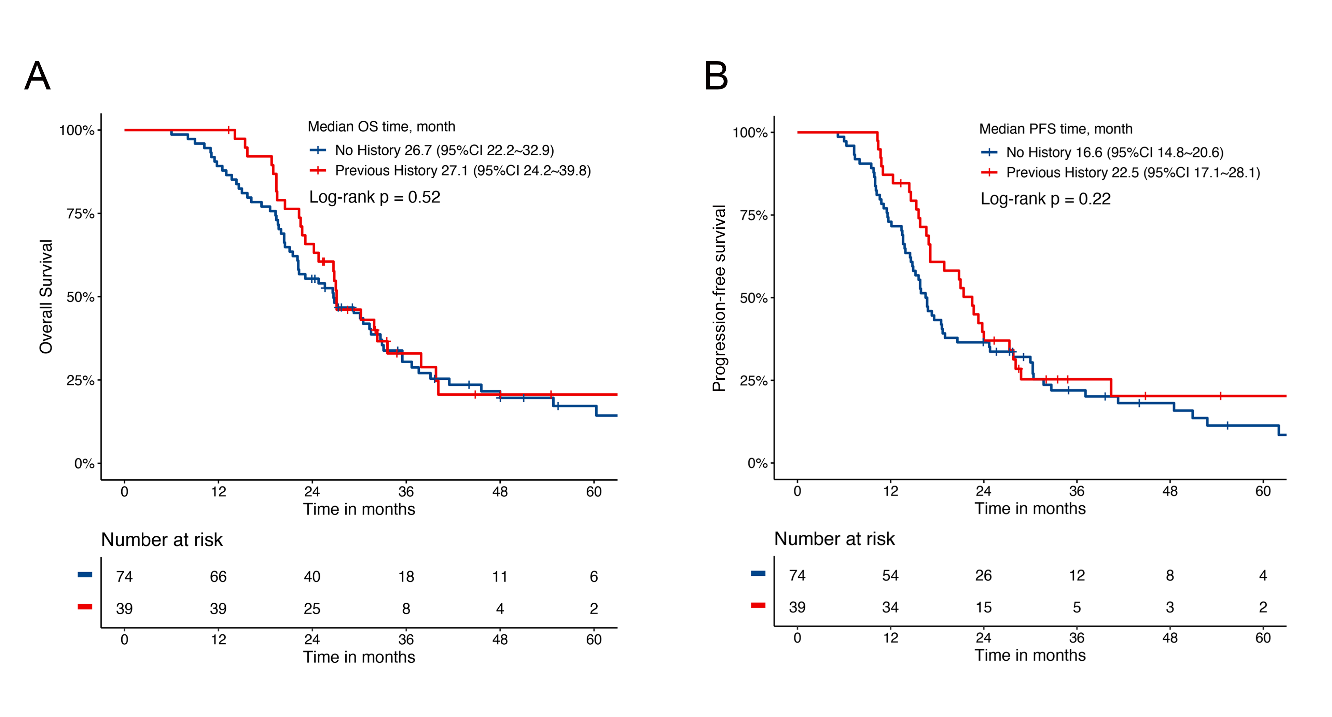


**Figure S1** Kaplan-Meier curve of median OS (A) and PFS (B) of patients with and without history of chemotherapy.


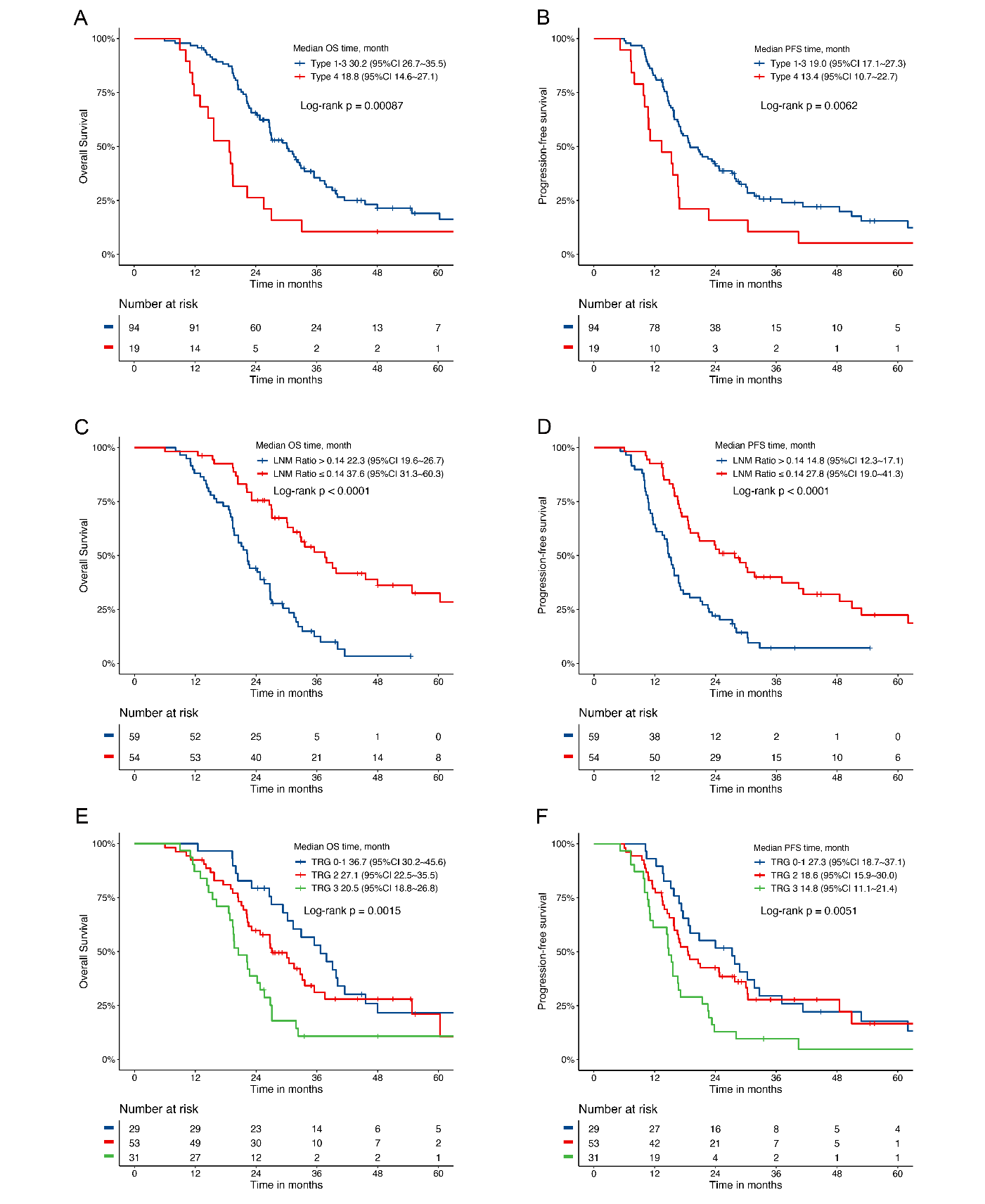


**Figure S2** Kaplan-Meier curve of median OS and PFS of patients with Borrmann type IV cancer and other types (A, B). Kaplan Meier curve of median OS and PFS of patients with LNM > 0.14 and LNM ≤ 0.14 (C**,** D). Kaplan-Meier curve of median OS and PFS of patients with TRG 0-1, 2 and 3 (E, F).


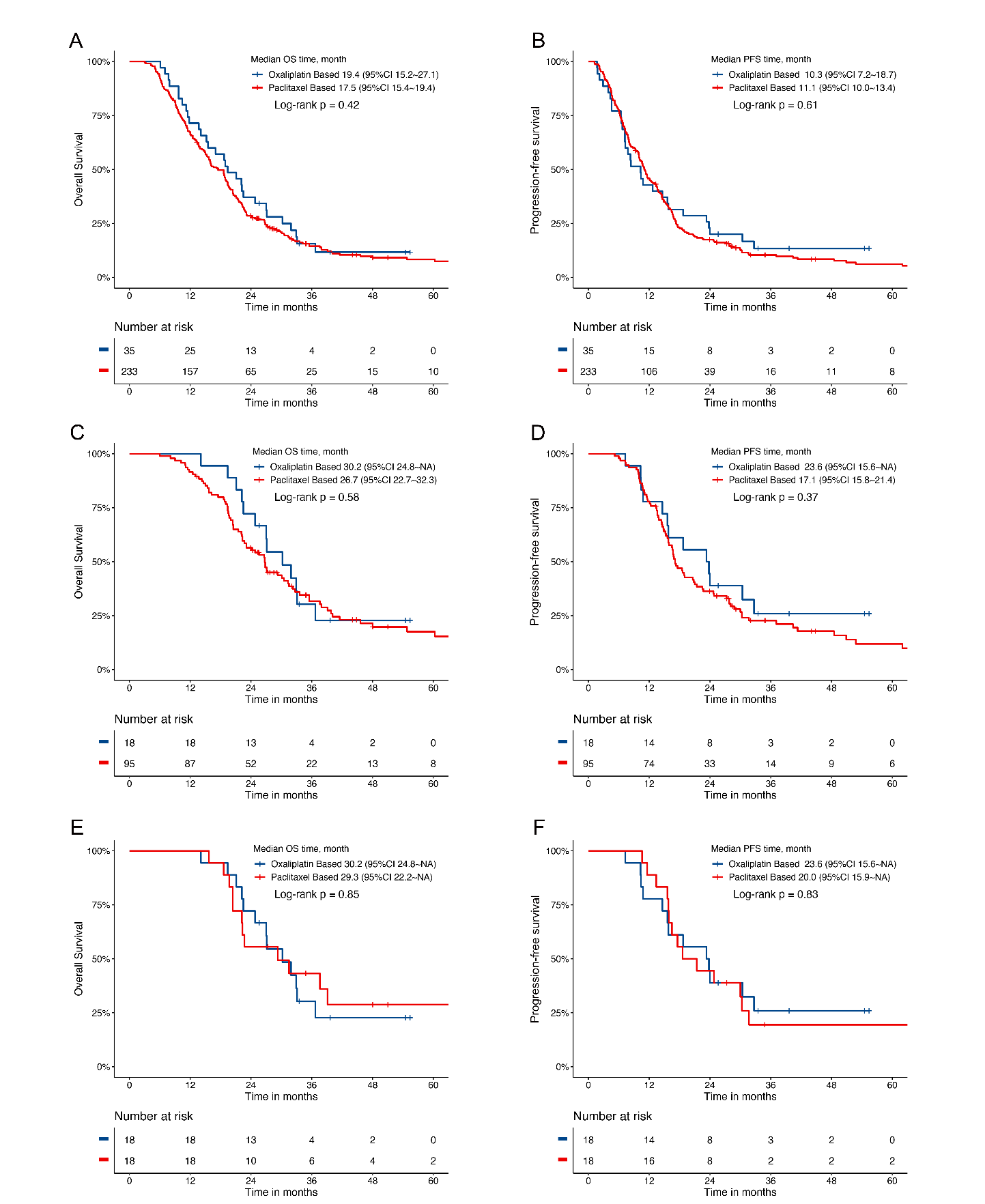


**Figure S3** Kaplan-Meier curve of median OS and PFS of patients in the entire group who underwent conversion therapy with the PS+ip PTX and SOX+ip PTX regimens (**A, B**). Kaplan-Meier curve of median OS and PFS of patients in the CS group who underwent conversion therapy with the PS+ip PTX and SOX+ip PTX regimens (**C, D**). Kaplan-Meier curve of median OS and PFS of patients in the CS group who underwent conversion therapy with the PS+ip PTX and SOX+ip PTX regimens after PSM (**E, F**).


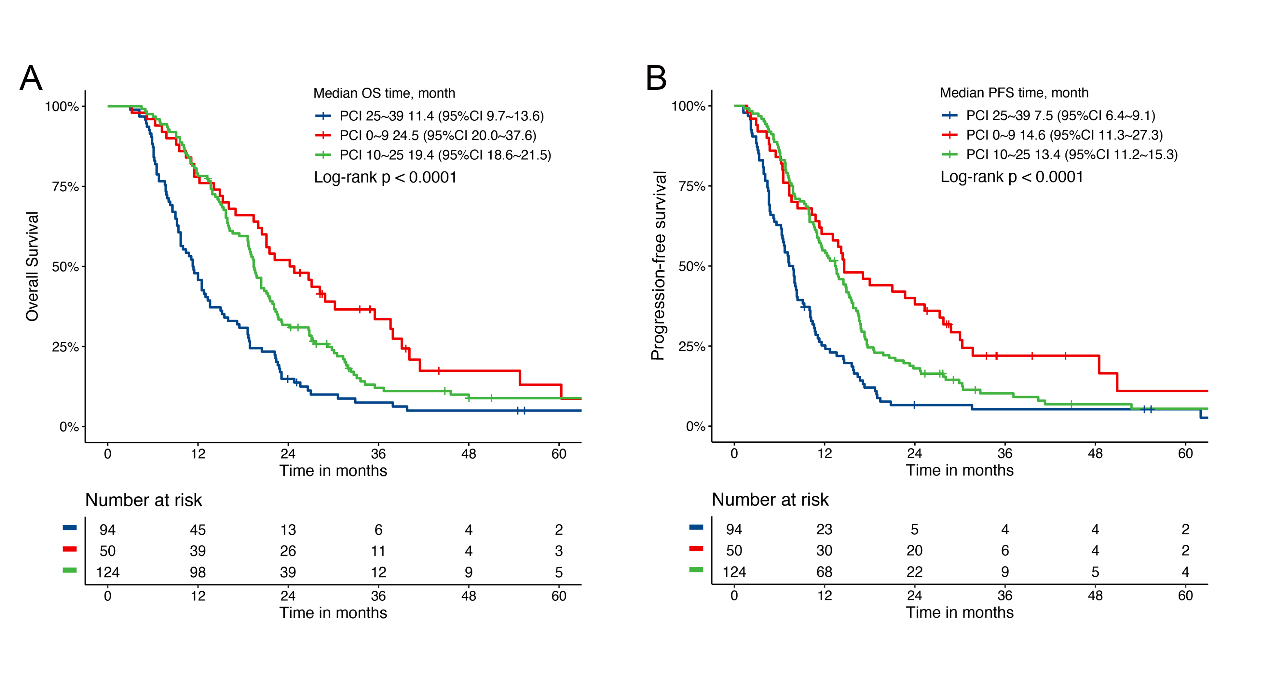


**Figure S4** Kaplan-Meier curve of median OS and PFS of patients with PCI 0-9, 10-25 and 25-39.


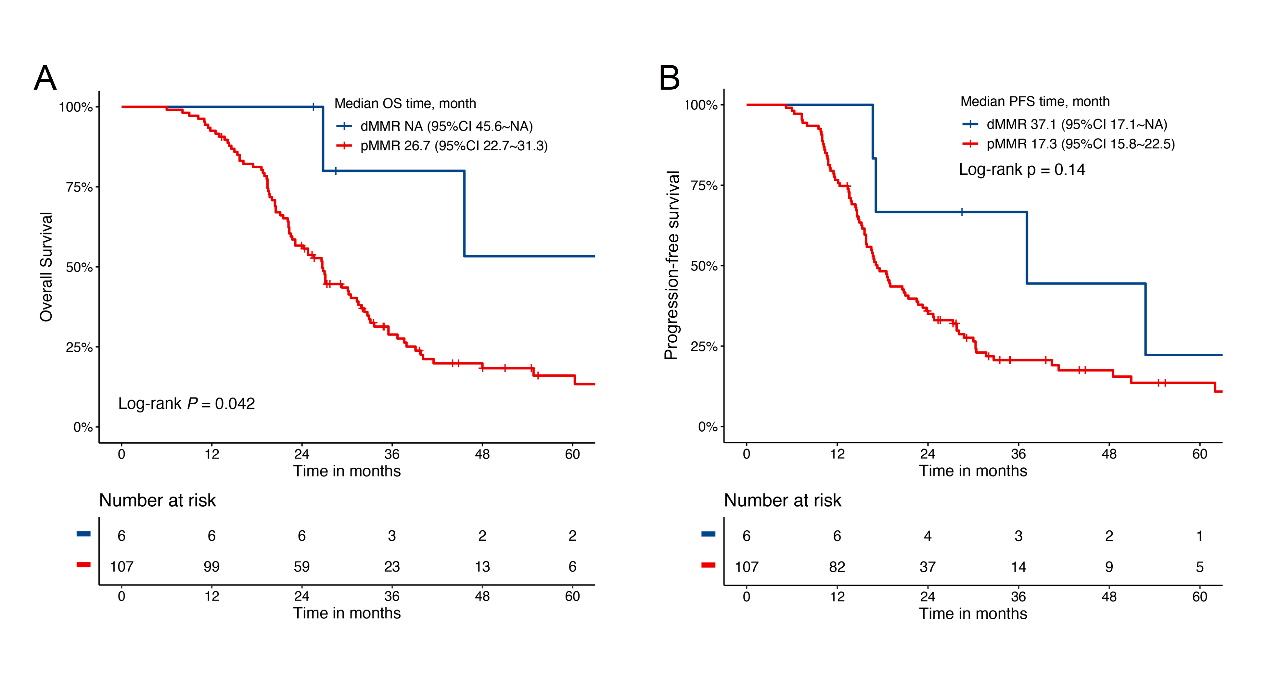


**Figure S5** Kaplan-Meier curve of median OS and PFS of patients with dMMR and pMMR (**A, B**).


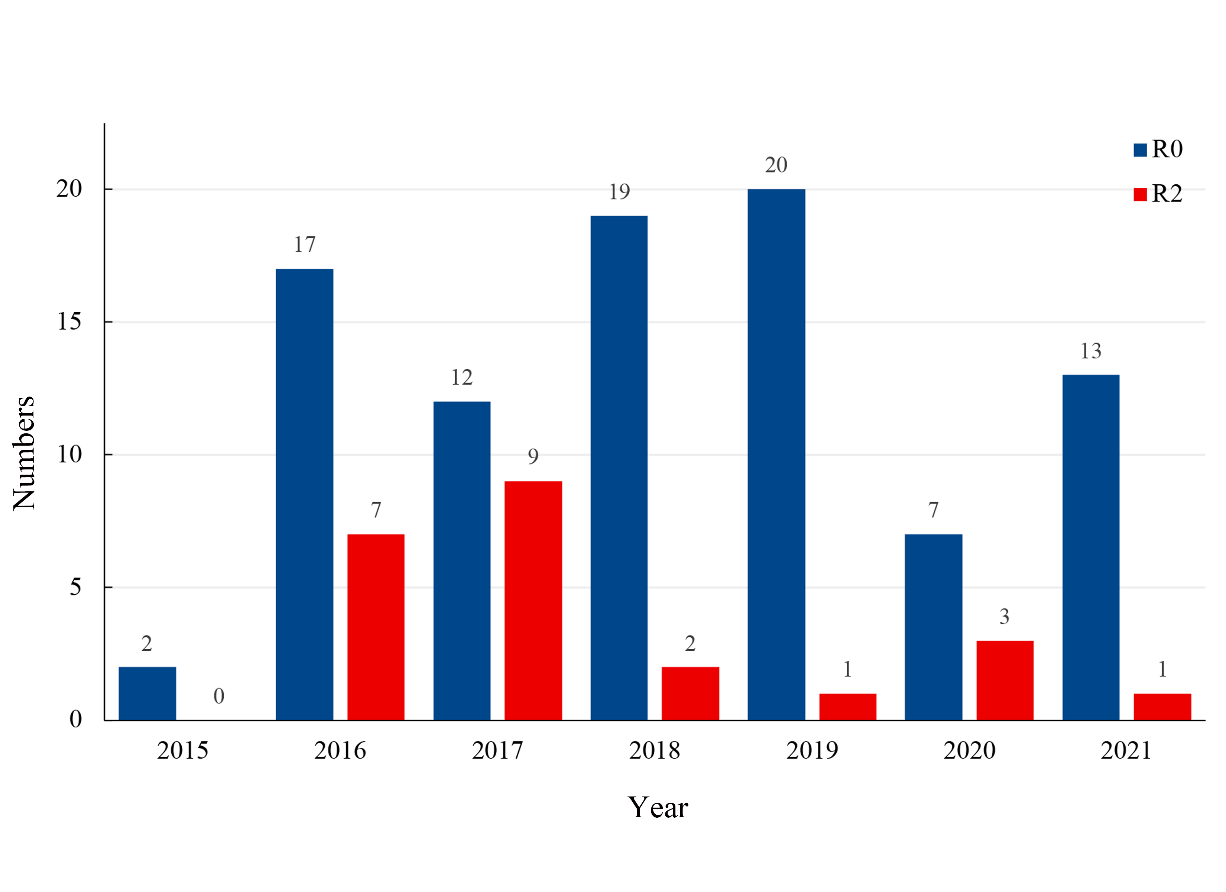


**Figure S6** The number of patients who underwent R0 and R2 resection from the year of 2015 to 2021 in our center.

**Table S1** Clinicopathological characteristics in patients undergo CS.

| **Variables** | **n** | **percent** |
| --- | --- | --- |
| Operation time |  | |
| Median, min | 240.0 (126.0-427.0) | |
| Estimated blood loss |  | |
| Median, ml | 60.0 (20.0-800.0) | |
| Length of postoperative hospital stay |  | |
| Median, day | 12.0 (7.0-82.0) | |
| Sex |  |  |
| Male | 43 | 38.1 |
| Female | 70 | 61.9 |
| Preoperative chemotherapy courses |  |  |
| < 6 | 47 | 41.6 |
| ≥ 6 | 66 | 58.4 |
| Type of gastrectomy |  |  |
| Distal | 33 | 29.2 |
| Total | 80 | 70.8 |
| Extent of resection |  |  |
| R0 | 90 | 79.6 |
| R2 | 23 | 20.4 |
| Combination of resection |  |  |
| Ovary | 23 | 20.4 |
| Gall bladder | 4 | 3.5 |
| Distal pancreas and spleen | 2 | 1.8 |
| Colon | 2 | 1.8 |
| Primary tumor location |  |  |
| U | 12 | 10.6 |
| M | 74 | 65.5 |
| L | 27 | 23.9 |
| Gross type |  |  |
| Borrmann I | 4 | 3.5 |
| Borrmann II | 6 | 5.3 |
| Borrmann III | 84 | 74.3 |
| Borrmann IV | 19 | 16.8 |
| Tumor size (cm) |  |  |
| ＜5.5 | 68 | 60.2 |
| ≥5.5 | 45 | 39.8 |
| Resected lymph nodes |  |  |
| ＜16 | 13 | 11.5 |
| ≥16 | 100 | 88.5 |
| Metastatic lymph node ratio |  |  |
| ≤0.14 | 54 | 47.8 |
| ＞0.14 | 59 | 52.2 |
| Pathological tumor stage (ypT) |  |  |
| ypT0-1 | 9 | 7.9 |
| ypT2 | 12 | 10.6 |
| ypT3 | 29 | 25.7 |
| ypT4 | 63 | 55.8 |
| Pathological nodal stage (ypN) |  |  |
| ypN0 | 25 | 22.1 |
| ypN1 | 19 | 16.8 |
| ypN2 | 21 | 18.6 |
| ypN3 | 48 | 42.5 |
| TRG |  |  |
| 0-1 | 29 | 25.7 |
| 2 | 53 | 46.9 |
| 3 | 31 | 27.4 |
| MMR status |  |  |
| dMMR | 6 | 5.3 |
| pMMR | 107 | 94.7 |

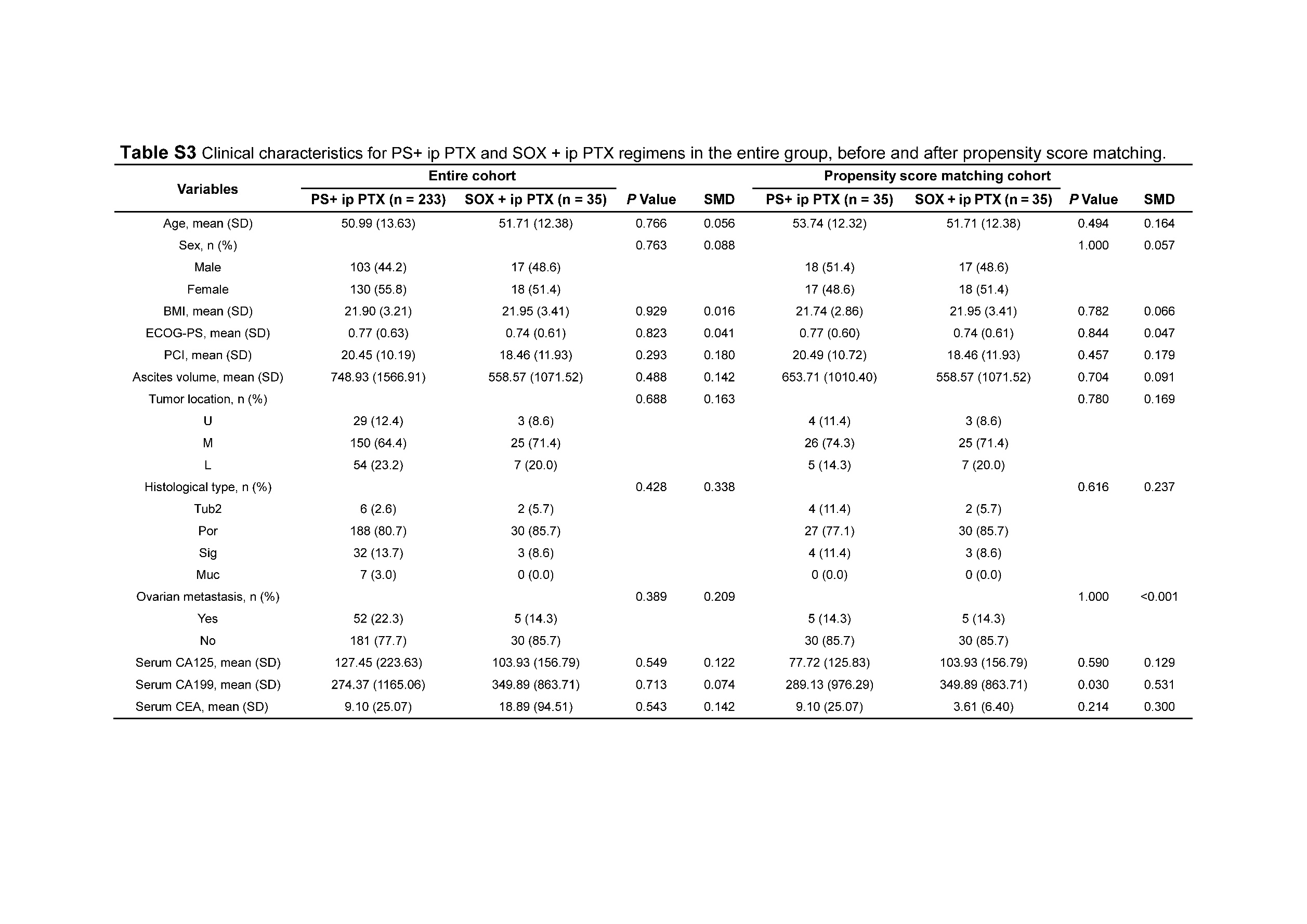


**Table S4** Multivariable analysis for female patients with or without ovariectomy in the NCS group.

| **Variables** | **HR** | **95% CI** | ***P* Value** |
| --- | --- | --- | --- |
| Age (year) |  |  |  |
| > 60 | 1.00 |  | 0.570 |
| ≤ 60 | 0.62 | 0.11 - 3.29 |  |
| BMI |  |  |  |
| > 20 | 1.00 |  | 0.517 |
| ≤ 20 | 0.73 | 0.28 – 1.19 |  |
| ECOG-PS |  |  |  |
| 0 - 1 | 1.00 |  | 0.624 |
| 2 | 0.59 | 0.07 -4.82 |  |
| P Stage |  |  |  |
| P1a/P1b | 1.00 |  | 0.180 |
| P1c | 0.15 | 0.01 – 2.39 |  |
| Amount of ascites, mL |  |  |  |
| ≤ 100 | 1.00 |  | 0.417 |
| > 100 | 0.60 | 0.17 – 2.06 |  |
| PCI |  |  |  |
| > 9 | 1.00 |  | 0.029 |
| ≤ 9 | 0.02 | 0.00 – 0.66 |  |
| Tumor location |  |  |  |
| L | 1.00 |  | 0.008 |
| U/M | 5.32 | 1.56 – 18.17 |  |
| Histological Type |  |  |  |
| tub2/por | 1.00 |  | 0.270 |
| sig/muc | 0.42 | 0.09 – 1.95 |  |
| Serum CA125, U/ml |  |  |  |
| > 47.4 | 1.00 |  | 0.978 |
| ≤ 47.4 | 1.02 | 0.34 – 3.07 |  |
| Serum CA19-9, U/ml |  |  |  |
| > 47.6 | 1.00 |  | 0.326 |
| ≤ 47.6 | 0.62 | 0.24 – 1.60 |  |
| Serum CEA, ng/ml |  |  |  |
| > 0.7 | 1.00 |  | 0.170 |
| ≤ 0.7 | 3.15 | 0.61 – 16.28 |  |
| Ovariectomy |  |  |  |
| With | 1.00 |  | 0.001 |
| Without | 13.34 | 3.02 – 58.98 |  |
| History of chemotherapy |  |  |  |
| With | 1.00 |  | 0.643 |
| Without | 1.31 | 0.42 – 4.15 |  |

**Table S5** Adjuvant chemotherapy regimens after conversion surgery.

| **Patient ID** | **First Line Adjuvant Regimen** | **Second Line Adjuvant Regimen** | **Third Line Adjuvant Regimen** | **Later Line Adjuvant Regimen** |
| --- | --- | --- | --- | --- |
| 1 | PS + ip PTX | S-1 + ip PTX | / | / |
| 2 | PS + ip PTX | / | / | / |
| 3 | PS + ip PTX | S-1 + ip PTX | PS | / |
| 4 | PS + ip PTX | S-1 + ip PTX | PS | S-1 |
| 5 | PS + ip PTX | / | / | / |
| 6 | PS + ip PTX | POS + ip PTX | FOLFIRI | / |
| 7 | PS + ip PTX | / | / | / |
| 8 | PS + ip PTX | S-1 + ip PTX | PS + ip PTX | CPT-11 |
| 9 | PS + ip PTX | / | / | / |
| 10 | PS + ip PTX | / | / | / |
| 11 | PS + ip PTX | S-1 + ip PTX | PS + ip PTX | CPT-11 |
| 12 | PS + ip PTX | S-1 + ip PTX | / | / |
| 13 | PS + ip PTX | / | / | / |
| 14 | S-1 + ip PTX | PS | S-1 | / |
| 15 | PS + ip PTX | S-1 + ip PTX | / | / |
| 16 | S-1 + ip PTX | PS + ip PTX | S-1 | / |
| 17 | PS + ip PTX | / | / | / |
| 18 | PS + ip PTX | PS | / | / |
| 19 | SOX + ip PTX | DS | CapeOX | / |
| 20 | PS + ip PTX | / | / | / |
| 21 | PS + ip PTX | CPT-11 | / | / |
| 22 | PS + ip PTX | / | / | / |
| 23 | PS + ip PTX | / | / | / |
| 24 | S-1 + ip PTX | PS + ip PTX | S-1 | / |
| 25 | PS + ip PTX | / | / | / |
| 26 | PS + ip PTX | CPT-11 | / | / |
| 27 | PS + ip PTX | / | / | / |
| 28 | PS + ip PTX | S-1 + ip PTX | S-1 | / |
| 29 | PS + ip PTX | / | / | / |
| 30 | PS + ip PTX | Cape + ip PTX | CapeOX + ip PTX | / |
| 31 | PS + ip PTX | CapeOX | / | / |
| 32 | PS + ip PTX | S-1 + ip PTX | PS + ip PTX | PS |
| 33 | S-1 + ip PTX | PS + ip PTX | / | / |
| 34 | PS + ip PTX | CapeOX | ip CDDP | / |
| 35 | S-1 + ip PTX | PS + ip PTX | S-1 + ip PTX | PS + ip PTX |
| 36 | PS + ip PTX | / | / | / |
| 37 | S-1 + ip PTX | PS + ip PTX | FOLFOX | / |
| 38 | S-1 + ip PTX | PS + ip PTX | FOLFOX | / |
| 39 | PS + ip PTX | CaoeOX | / | / |
| 40 | S-1 + ip PTX | / | / | / |
| 41 | S-1 + ip PTX | S-1 | / | / |
| 42 | PS + ip PTX | / | / | / |
| 43 | PS + ip PTX | / | / | / |
| 44 | S-1 + ip PTX | PS + ip PTX | PS + ip PTX + ip CDDP | / |
| 45 | PS + ip PTX | S-1 + ip PTX | PS + ip PTX | AP |
| 46 | PS + ip PTX | S-1 + ip PTX | Cape + ip PTX | / |
| 47 | PS + ip PTX | POS + ip PTX | / | / |
| 48 | PS + ip PTX | / | / | / |
| 49 | PS + ip PTX | S-1 + ip PTX | PS + ip PTX | CPT-11 |
| 50 | S-1 + ip PTX | PS + ip PTX | / | / |
| 51 | PS + ip PTX | S-1 + ip PTX | PS + ip PTX | / |
| 52 | PS + ip PTX | / | / | / |
| 53 | PS + ip PTX | S-1 + ip PTX | PS + ip PTX | CapeOX —> FOLFIRI |
| 54 | PS + ip PTX | S-1 | SOX + ip PTX | / |
| 55 | PX + ip PTX | S-1 + ip PTX | / | / |
| 56 | S-1 + ip PTX | SOX + ip PTX | S-1 + ip PTX | / |
| 57 | S-1 + ip PTX | SOX + ip PTX | S-1 + ip PTX | S-1 + ip PTX + ip CDDP |
| 58 | PS + ip PTX | / | / | / |
| 59 | PS + ip PTX | FOLFIRI + ip PTX + ip CDDP | / | / |
| 60 | PS + ip PTX | PS | / | / |
| 61 | SOX + ip PTX | PS + ip PTX | / | / |
| 62 | S-1 + ip PTX | S-1 | / | / |
| 63 | S-1 + ip PTX | DOX + DOC IP | PS + ip PTX | FOLFOX —> CPT-11 —> PD-1 |
| 64 | SOX + ip PTX | S-1 + ip PTX | / | / |
| 65 | PS + ip PTX | PS + ip PTX + ip CDDP | / | / |
| 66 | SOX + ip PTX | PS + ip PTX | S-1 + ip PTX | / |
| 67 | PS + ip PTX | nab-PS + ip nab-PTX + ip CDDP | PS + ip PTX | / |
| 68 | PS + ip PTX | SOX + ip PTX | / | / |
| 69 | PS + ip PTX | / | / | / |
| 70 | PS + ip PTX | PS + ip PTX + PD-1 | / | / |
| 71 | PS + ip PTX | / | / | / |
| 72 | SOX + ip PTX | / | / | / |
| 73 | PS + ip PTX | S-1 + ip PTX | CapeOX | nab-PTX + ip nab-PTX —> ip PTX |
| 74 | S-1 + ip PTX | PS + ip PTX | / | / |
| 75 | PS + ip PTX | S-1 + ip PTX | ip PTX + ip CDDP | / |
| 76 | PS + ip PTX | / | / | / |
| 77 | PS + ip PTX | PS | / | / |
| 78 | PS + ip PTX | S-1 + ip PTX | / | / |
| 79 | PS + ip PTX | S-1 + ip PTX | PS-P | S-1 |
| 80 | PS + ip PTX | / | / | / |
| 81 | PS + ip PTX | PS + ip PTX + ip CDDP | CapeOX-P + ip PTX | / |
| 82 | PS + ip PTX | / | / | / |
| 83 | S-1 + ip PTX | PS + ip PTX | CapeOX | Cape-H —> Cape-H + ip PTX  —> Cape-H + ip PTX + ip CDDP  —> H + ip PTX + ip CDDP + PD-1 |
| 84 | S-1 + ip PTX | SOX + ip PTX | S-1 + ip PTX | PS + ip PTX + ip CDDP —> PD-1 |
| 85 | PS + ip PTX | DX | RegoNivo | / |
| 86 | PS + ip PTX | nab-PS-AP + ip nab-PTX | PD-1 | / |
| 87 | PS + ip PTX | S-1 + ip PTX | / | / |
| 88 | PS + ip PTX | S-1 + ip PTX | PS + ip PTX | / |
| 89 | PS + ip PTX | S-1 + ip PTX | / | / |
| 90 | SOX + ip PTX | / | / | / |
| 91 | PS + ip PTX | / | / | / |
| 92 | SOX + ip PTX | / | / | / |
| 93 | PS + ip PTX | / | / | / |
| 94 | PS + ip PTX | PS + ip PTX + ip CDDP | PD-1 | / |
| 95 | SOX + ip PTX | / | / | / |
| 96 | SOX + ip PTX | S-1 + ip PTX | S-1 | / |
| 97 | nab-PS + ip PTX | ip PTX | / | / |
| 98 | SOX + ip PTX | S-1 + ip PTX | PD-1 + ip PTX | / |
| 99 | S-1 + ip PTX | / | / | / |
| 100 | PS + ip PTX + ip CDDP | PS + ip PTX | CapeOX-P | / |
| 101 | PS + ip PTX | PS + ip PTX + ip CDDP | PS + ip PTX + ip CDDP + PD-1 | / |
| 102 | PS + ip PTX | / | / | / |
| 103 | PS + ip PTX | PS + ip PTX + ip CDDP | / | / |
| 104 | PS + ip PTX | PS + ip PTX + ip CDDP + PD-1 | / | / |
| 105 | PS + ip PTX | S-1 + ip PTX | / | / |
| 106 | PS + ip PTX | S-1 + ip PTX | CapeOX + ip PTX + PD-1 | Cape + ip PTX + ip CDDP + PD-1  —> CPT-11 —> Lenvatinib + PD-1 |
| 107 | PS + ip PTX + ip CDDP | / | / | / |
| 108 | nab-PS + ip nab-PTX | nab-PS + ip nab-PTX + PD-1 | S-1 + ip nab-PTX | nab-PS + ip nab-PTX + ip CDDP |
| 109 | S-1 + ip PTX | PS + ip PTX | S-1 + ip PTX | / |
| 110 | PS + ip PTX | / | / | / |
| 111 | SOX + ip PTX | S-1 + ip PTX | / | / |
| 112 | PS + ip PTX + ip CDDP | / | / | / |
| 113 | PS + ip PTX | / | / | / |
